# Origami-Inspired Frequency Selective Surface with Fixed Frequency Response under Folding

**DOI:** 10.3390/s19214808

**Published:** 2019-11-05

**Authors:** Deanna Sessions, Alexander Cook, Kazuko Fuchi, Andrew Gillman, Gregory Huff, Philip Buskohl

**Affiliations:** 1 Department of Electrical Engineering, Pennsylvania State University, University Park, PA 16801, USA; ghuff@psu.edu; 2 NextFlex, San Jose, CA 95131, USA; acook@nextflex.us; 3 University of Dayton Research Institute, Dayton, OH 45469, USA; kfuchi1@udayton.edu; 4 Aerospace Systems Directorate, Air Force Research Laboratory, WPAFB, OH 45433, USA; 5 Materials and Manufacturing Directorate, Air Force Research Laboratory, WPAFB, OH 45433, USA; andrew.gillman.2@us.af.mil (A.G.); philip.buskohl.1@us.af.mil (P.B.)

**Keywords:** Archimedean spiral, circular polarization, direct write, frequency selective surfaces, Miura-ori, origami

## Abstract

Filtering of electromagnetic signals is key for improved signal to noise ratios for a broad class of devices. However, maintaining filter performance in systems undergoing large changes in shape can be challenging, due to the interdependency between element geometry, orientation and lattice spacing. To address this challenge, an origami-based, reconfigurable spatial X-band filter with consistent frequency filtering is presented. Direct-write additive manufacturing is used to print metallic Archimedean spiral elements in a lattice on the substrate. Elements in the lattice couple to one another and this results in a frequency selective surface acting as a stop-band filter at a target frequency. The lattice is designed to maintain the filtered frequency through multiple fold angles. The combined design, modeling, fabrication, and experimental characterization results of this study provide a set of guidelines for future design of physically reconfigurable filters exhibiting sustained performance.

## 1. Introduction

Physically reconfigurable electromagnetic (EM) devices undergo shape change to attain electromagnetic performance tuning or tailored structural properties. The former is a type of passive EM signal control achieved through physical rearrangements of active EM components. In the latter, the primary motivation of the shape change comes from considerations other than EM functionality, such as structural deformations for deployable or adaptive structures, wearable devices, and conformal filters, while the EM performance is expected to remain fixed. In particular, exploration of EM device designs that maintain their functionality throughout a large structural deformation is identified to be one of the critical areas of scientific research according to the 2016 Multidisciplinary University Research Initiative solicitation entitled “4D Electromagnetic Origami” by the Air Force Office of Scientific Research; however, limited investigations on this subject are seen in literature. This article introduces a novel design of frequency selective surface (FSS) that combines a well-studied origami pattern called Miura-ori and densely packed circularly polarized four-arm Archimedean spirals to achieve a deployable structure that maintains frequency response. To the best of the authors’ knowledge, this is the first study on an origami-inspired physically reconfigurable EM device targeted at fixed frequency response.

Physically reconfigurable EM designs inspired by the intricate art of origami generate complex, functionally relevant 3D surfaces from easily manufactured 2D sheets, while being simply described by a small number of parameters within well-established mathematics. The tuning of EM performance through folding has been previously demonstrated in frequency selective surfaces (FSSs) [1,2,3] and antenna [4,5,6,7,8] applications. For example, linearly polarized FSSs using dipoles and the Miura-ori folding pattern offer frequency tuning through relative re-arrangement [1], relative positioning, and folding dipole elements [2,3] exhibiting frequency shifts on the order of 200 MHz for a 36° fold increase in [2] and 1 GHz for a 60° fold increase for a single layer FSS in [3]. However, there are few examples of the opposite case: Electromagnetic devices that maintain EM performance frequency while undergoing physical reconfiguration, particularly via folding. Devices with these criteria are of interest for flexible and conformal electronics applications, where decreased size, weight, and integrated functionality are important. Application spaces may include antenna arrays and FSSs embedded in adaptive wings where the structure changes due to mechanical constraints or deployable EM structures such as radomes or FSSs.

The conductive spiral trace shape and origami pattern of the FSS were selected for consistent frequency response over a wide range of polarization angles and deformations. Miura-ori is an origami pattern that reduces to a highly compact state when folded [9] and provides diverse deformations in intermediate folded states involving in-plane folding, bending and twisting [10]. These deformations can be tailored to produce a folded surface conforming to an arbitrary curvature. In addition, the facets may be constructed using a fairly rigid material and be subject to slight bending or twisting; most local deformations occur along the folds. This combination of structural characteristics make this an advantageous folding pattern for mechanical designs in large scale reconfigurable systems.

The fold repeatability and structural integrity of origami devices have been addressed using various materials that ease challenges in folding and flexibility. This includes bridging EM elements over fold lines [3] to mitigate challenges with folding and flexibility of metallic components and using shape memory alloys [11] and polymers [12,13] for fold actuation. These strategies can be directly applied to future implementation of this work with a rugged substrate design for a specific structural application but are out of the scope of this study.

Spiral elements are circularly-polarized [14,15] and are utilized in this study to mitigate detuning and response degradation caused by angular and polarization dependency, observed in linearly-polarized elements on folded surfaces [1,2]. A densely packed spiral lattice is printed on each facet of the Miura-ori pattern, to further reduce the angular dependency. An advanced additive manufacturing technique is employed to fabricate this device for an X-band application, with a high level of precision. The origami FSS is fabricated through extrusion printing of a polyurethane-based, silver ink [16,17] on a polypropylene (PP) substrate. The PP sheet is laser-scored along fold lines prior to spiral printing and folded into a Miura-ori pattern post-printing. The following includes details of the design, simulation, fabrication method and the experimental characterization of the conformal Miura FSS device.

## 2. Design of the Conformal FSS

**Figure 1:**
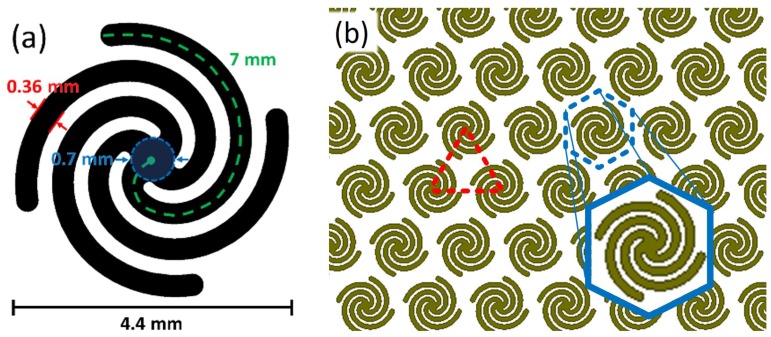
Spiral-based frequency selective surface (FSS) design with triangular packing. Schematic of (**a**) Archimedean spiral dimensions, (**b**) triangular lattice of spirals including unit cell for periodic simulation (blue).

Figure 1a shows a schematic of the four-armed Archimedean spiral used in this work [14]. Each arm is constructed based on the expressions in Equation (1) and thickened to obtain width *w*. The arm is capped at the end by a semi-circle of diameter *w* to match the arm width, and a circle of radius *r* is inserted at the center where the four arms converge. In this work, a=0.424 and $b=\{0,\;\frac{\pi }{2},\;\pi ,\;\frac{3\pi }{2}\}$. The number of turns in each spiral arm are 0.80. These parameters result in 4.4 mm diameter spiral elements.

(1) $$\begin{array}{c} X=at\cos(t+b)\\ Y=at\sin(t+b)\\ -2\pi <t<2\pi \\ b=0,\frac{\pi }{2},\pi ,\frac{3\pi }{2} \end{array}$$

The spirals are arranged into a lattice with 5 mm center-to-center spacing. The spirals are tessellated to fill each parallelogram facet of a Miura-ori origami pattern. The Miura-ori fold pattern lends well to triangular and hexagonal packing when the parallelogram facets are rhombic with 60° vertex angles which allows for elements to have equilateral triangle spacing between centers. This triangular lattice is the tightest packing possible for the spiral elements on the Miura-ori substrate. Two lattices were explored in simulation and fabrication, a densely packed triangular lattice and a loosely packed hexagonal lattice. Figure 1b shows a triangular lattice and its hexagonal unit cell. In this work, samples are fabricated with a triangular lattice on the facets and a hexagonal lattice at the fold lines to accommodate folding.

**Figure 2:**
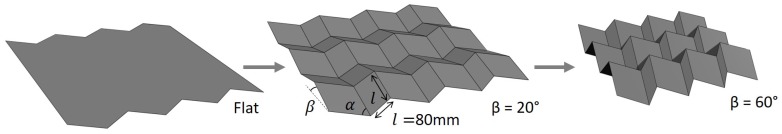
Miura-ori based FSS design. Demonstrating intermediate fold angles of β = 0°, 20°, and 60°, corresponding projected area change of AAo = 1, 0.94, 0.50.

Figure 2 shows the folding progression of the Miura-ori fold pattern. Miura-ori unit cells consist of four parallelogram facets tessellated into a doubly corrugated pattern. The fabricated samples in this work are comprised of a sheet of polypropylene containing a grid of 7 × 5 parallelograms each with vertex angle α = 60°, fold angle β, and side length l=80 mm.

## 3. Design Study

A triangular lattice is simulated using Floquet ports in Ansys HFSS to create a periodic structure based on the unit cell shown in Figure 1b. The lattice is simulated on a 0.75 mm thick polypropylene (PP) substrate ($\epsilon_{r}=2.2,\tan\delta =0.01$). Spirals are assigned conductivity corresponding to measured values of the conductive ink (22,500 Scm). Ink formulation is explained more in Section 4. Additional studies of material conductivity impact on RF performance are found in Appendix A.4.

**Figure 3:**
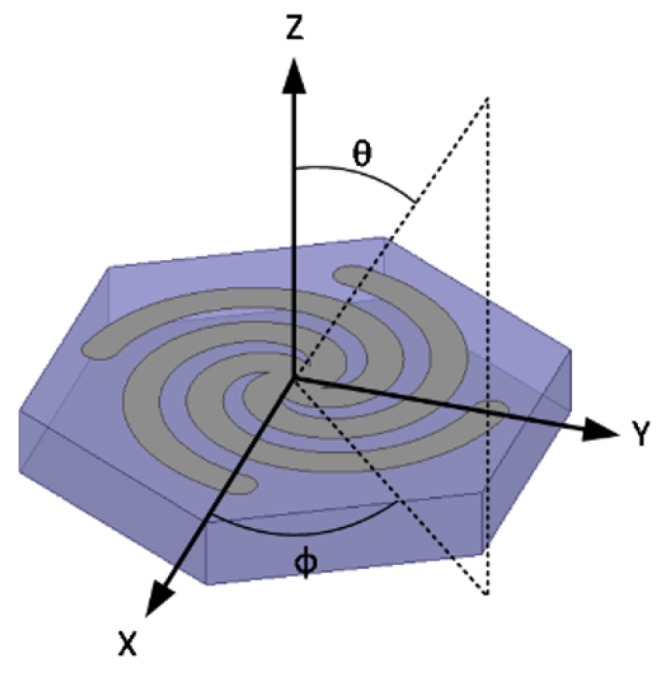
Representative coordinate system denoting the incoming electromagnetic (EM) signal orientation and used in the oblique incidence (θ) and polarization (ϕ) simulation sweeps.

The lattice is subjected to incident plane waves at normal and oblique incidence. Figure 3 shows the corresponding coordinate system for oblique incidence sweeps. The oblique incidence results can be extrapolated to predict the impact folding Miura-ori facets will have on the spiral lattice frequency response. However, it should be noted there are additional scattering parameters in the origami geometry not present in this model.

**Figure 4:**
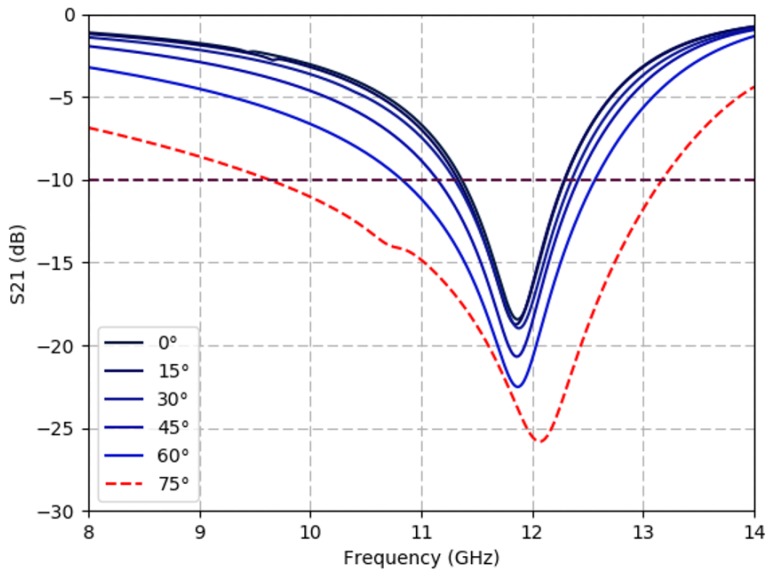
Spiral-based FSS specificity diminishes at large angles of incidence. Simulated $S_{21}$ response of a triangular lattice varying oblique incidence values, $\theta =0^{\circ },15^{\circ },30^{\circ },45^{\circ },60^{\circ },75^{\circ }$, with constant ϕ=0° and in the flat state (β=0°).

**Figure 5:**
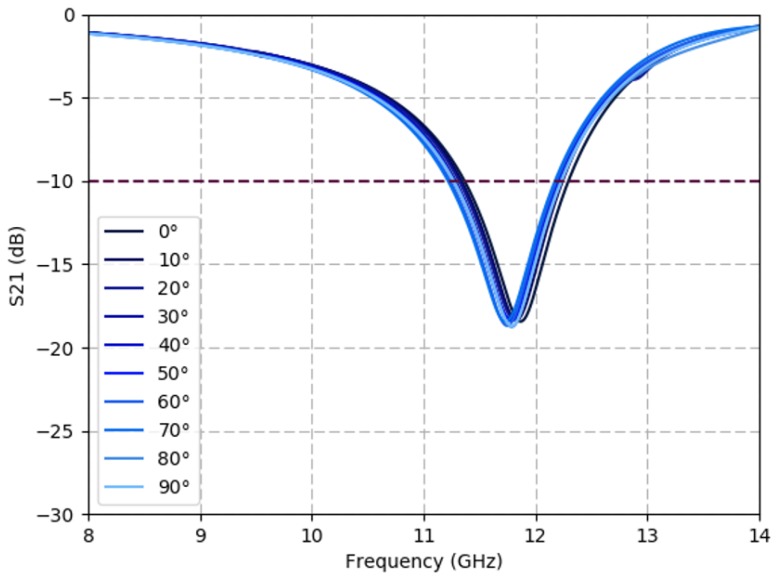
Spiral-based FSS demonstrates polarization independence. Simulated $S_{21}$ response of a triangular lattice under varying polarization angle values (ϕ) with constant incidence angle (θ=0°) in the flat configuration (β=0°).

Figure 4 and Figure 5 show simulation results from the angle of incidence and polarization studies. Figure 4 shows the simulated transmission response ($S_{21}$) of a triangular spiral lattice while varying the incident angle (θ). The frequency response of the lattice remains relatively constant through varying the incident angle of the plane wave up to θ=30°. Filtering bandwidth increases for incidence angles greater than θ=45°, from 1040 MHz at θ=30° to 1730 MHz at θ=60°; however, resonance is maintained at around 11.8 GHz (between 11.74 GHz and 11.87 GHz). At θ=75° we observe a rightward shift in center frequency to 12.07 GHz and a significant broadening of the bandwidth of the filter (3.54 GHz). At θ=90° (grazing angle) the device is non-functional, as would be expected for this planar geometry. The incidence angle parameter sweep on a flat substrate (β=0°) serves as an approximation for the transmission behavior of a folded substrate, as the facet orientations will alter the effective incidence of the incoming EM signal. The results of Figure 4 suggest filtering performance should be stable for a fold angle range of β=0° and 60°.

Figure 5 shows the simulated $S_{21}$ response of a triangular spiral lattice while varying polarization of the incident wave. Due to the four-fold symmetry of the spiral geometry and the densely packed FSS design, the frequency response remains constant despite varying polarization at any $\phi \in [0^{\circ },360^{\circ }]$, while tunable FSSs based on linear elements in previous works [2,3] are operational at two orthogonal linear polarization directions only. The lattice maintains its center frequency and its bandwidth through all polarization angles. This is to be expected as the spiral geometry is designed to be polarization independent.

All results imply that the spiral lattice is suited to maintain a notch filter at a constant frequency with an $S_{21}$ below −10 dB for fold angles ranging from β=0° to 60°. These results can be extrapolated to infer how the response of the spirals will be impacted by the origami folds of the structure at varying fold angles.

## 4. Fabrication Method

**Figure 6:**
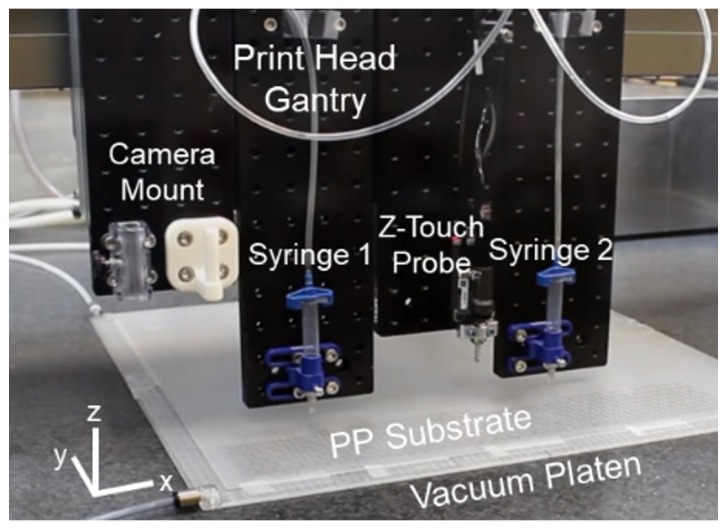
Origami FSS fabrication setup. Image of Aerotech gantry print head with camera mount, touch probe and dual syringe attachments. A custom vacuum platen was employed to improve uniformity in the z-height of the polypropylene (PP) substrate.

An extrusion print method was employed for sample fabrication. This method produces spirals with high repeatability while also allowing for rapid translation from design to print. Conductive ink is dispensed via pressure driven syringe. The print path is controlled directly via g-code or Python code to facilitate higher levels of control over the print process. Prior to printing the spiral traces, the printer measures the height of the substrate at each spiral position and then adjusts the height of the syringe tip to accommodate for the variances in the thickness and flatness of the polypropylene. This process results in accuracy on the order of 1 μm. Figure 6 shows the gantry printer setup. The impact of the print process on spiral geometries is addressed in Appendix A.

A silver thermoplastic polyurethane (Ag-TPU) ink formulation [16] was used to print the conductive traces, consisting of a 40:60 volume ratio of silver flakes (procured from Inframat Inc) to TPU (BASF Elastollan) dispersed in N-methyl-2-pyrrolidone (NMP). This ink was used for its flexibility, stability, and repeatability in the extrusion print process. Importantly, it can adhere to a multitude of materials, including the polypropylene used in this work.

**Figure 7:**
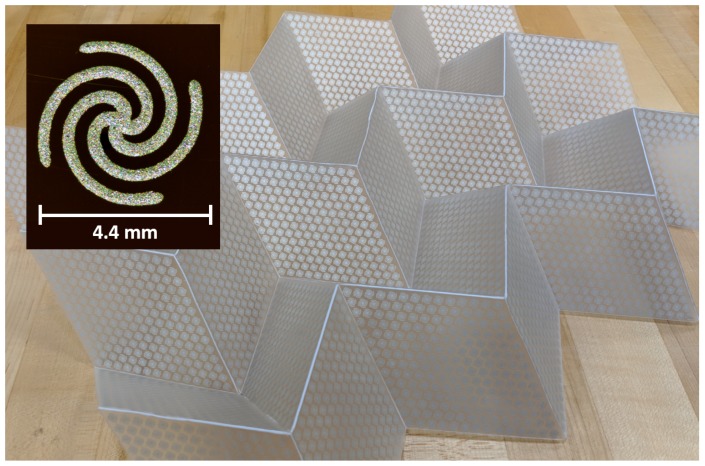
Fabricated specimen Miura-ori FSS. Triangular lattice of silver thermoplastic polyurethane (Ag-TPU) spirals printed on a laser-cut PP substrate folded according to the Miura-ori pattern. Inset: Representative optical image of the printed Ag-TPU spirals.

Prior to printing the Ag-TPU ink on the PP substrate, the PP was laser-scored with the Miura-ori fold pattern. The spiral traces were then printed in Ag-TPU ink on the flat (unfolded) substrate using two syringes printing two spirals at once to increase print throughput. Post-printing, the sample was annealed at 125 °C in an oven to evaporate the residual NMP solvent, leaving behind silver particles encased in TPU. Figure 7 shows a fabricated prototype of this Miura-ori spiral FSS with a triangular lattice.

## 5. Experimental Setup

The origami FSSs are tested in a free-space setup with the sample placed between an incident horn and a receive horn. The test setup is calibrated using a gated-reflect-line (GRL) technique to mitigate the effects of external signals in the testing area. An 8 ns time gate was applied to the measurements. This setup measures the impact of the origami FSS acting as a spatial filter by comparing the incident and received field at the two horns.

**Figure 8:**
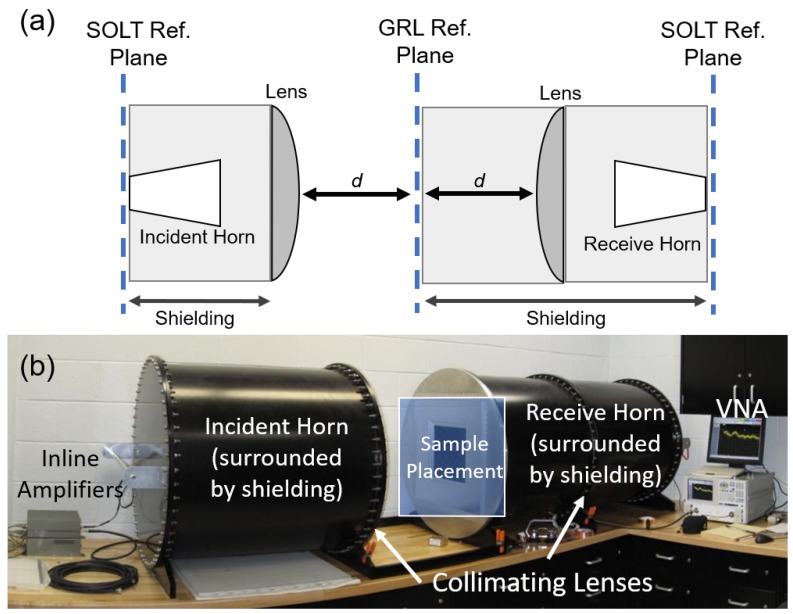
Experimental setup utilizing free-space testing and gated-reflect-line (GRL) calibration. (**a**) Schematic of FSS test setup denoting horns, the short-open-load-thru (SOLT) calibration planes and the GRL plane. (**b**) Image of experimental setup, highlighting the horn shielding apparatus.

As pictured in Figure 8, the setup consists of two 2–18 GHz horn antennas encased in a shielding barrel to obtain an acceptable noise floor for testing. The beams of these horns are collimated with RF lenses to focus the spot size at the GRL reference plane where the sample resides. This setup can accommodate samples up to 24 × 24 inches with a maximum thickness of 0.375 inches.

The FSS is subjected to deformations through folding and its spatial filtering impact is measured at each folded state. The FSS is folded by hand and the distance between mountain folds are measured to verify a precise fold angle. The structure is held in place by a test fixture between the two horn antennas. Due to the polarization independence exhibited by the spiral elements, the samples were tested under a horizontally polarized, normal incidence wavefront after determining the vertically polarized case maintained comparable results at all fold angles.

## 6. Experimental Results

**Figure 9:**
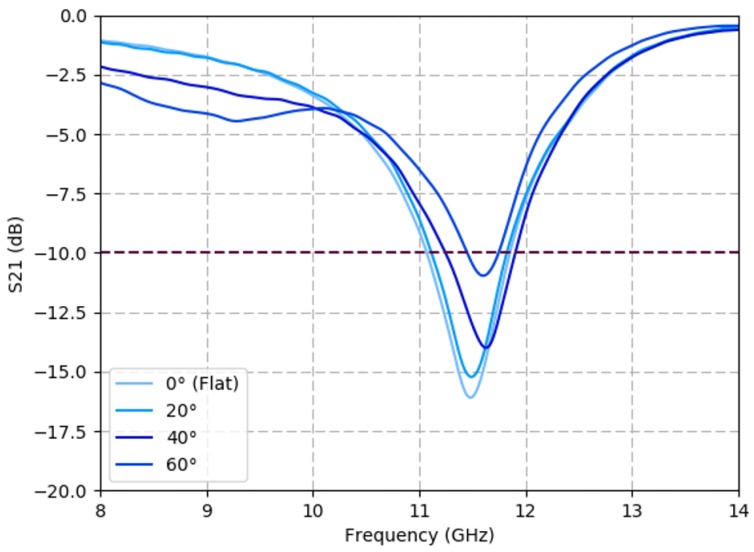
Miura-ori FSS maintains filtering performance under folding. Experimental transmission coefficient (S21) results of a Miura-ori spiral FSS, with a triangular lattice, at various fold states ranging from β=0° (flat) to 60°. The operational folding range is equivalent to a 50% decrease in FSS area.

A Miura-ori spiral FSS was tested using the experimental setup described in Section 5. Figure 9 shows the frequency response over the X-band (8–12 GHz) for the sample at multiple fold angles.

These results show that the origami FSS that maintains a fixed frequency response through a large shape deformation when compared to previous origami studies using dipoles. The structure maintains an acceptable filtering response below –10 dB at 11.5 GHz from a flat state to a 60° fold. The measurements agree with the incidence angle simulations from Figure 4, which predicted consistent filtering frequency up to θ=30° (β=30°) and a rightward frequency shift at higher incidence angles. However, deviances due to test setup and sample fabrication should be noted. The center frequency of the filter differs by approximately 250 MHz between the simulated (11.80 GHz) and measured (11.55 GHz) average results. The slight quantitative deviation of the center frequency is likely the consequence of variances in printed spiral geometry and the conductivity of the Ag-TPU ink, which is dependent on ink formulation and the sintering processes. The geometric and material property variances are further discussed in studies found in Appendix A. The trend of the measured FSS shows a decrease in filtered $S_{21}$ response as fold angles increase, this is attributed to many spirals folding out of plane during the experiment thereby changing the number of spirals in the GRL measurement space. Additionally, spirals are simulated in an infinite plane rather than a full Miura-ori unit cell due to computational limitations in simulating 784 spirals per each 4-facet unit cell. This can impact the simulated effects of angles of incidence on measured $S_{21}$ values.

## 7. Conclusions

An origami-based FSS demonstrating consistent stop-band spatial filtering under large deformation was designed, fabricated and experimentally demonstrated in this study. Through systematic simulations, the geometry and close packing of the spirals demonstrated robust tolerance to changes in polarization and incident angles. The Miura-ori FSS leverages this quality to maintain filter performance at a fixed frequency, while undergoing a 50% decrease in size. The FSS design concepts from this study provide guidelines that can be applied to areas requiring physical reconfiguration, such as aerospace systems, wearables, and satellite operations.

## Figures and Tables

**Figure A1:**
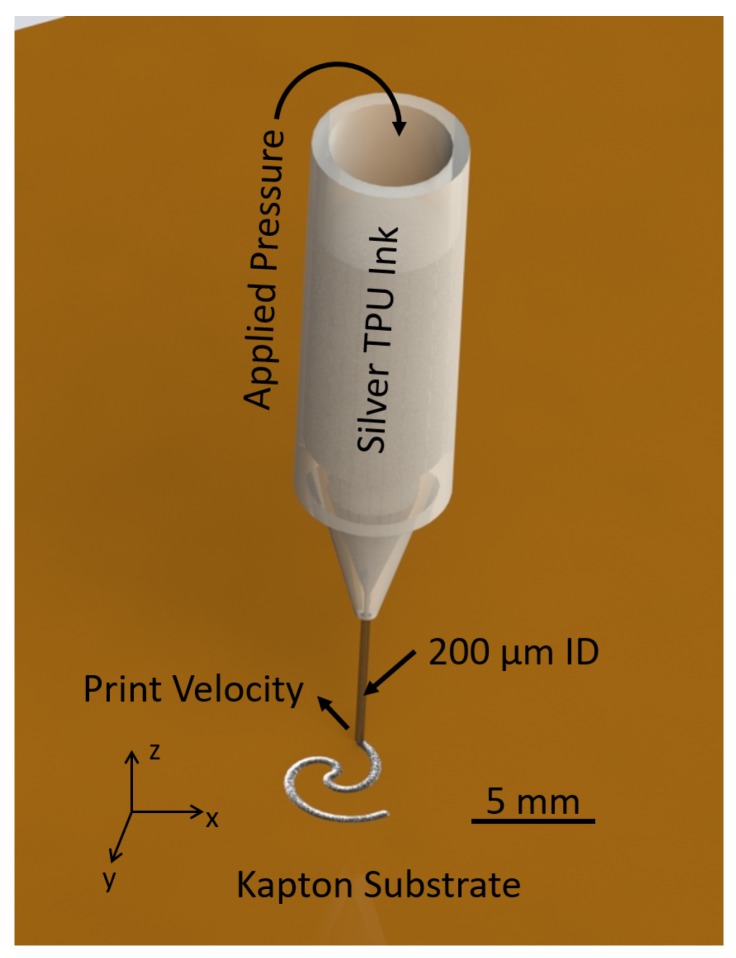
Print setup denoting syringe placement and size for extrusion direct-ink write process.

**Figure A2:**
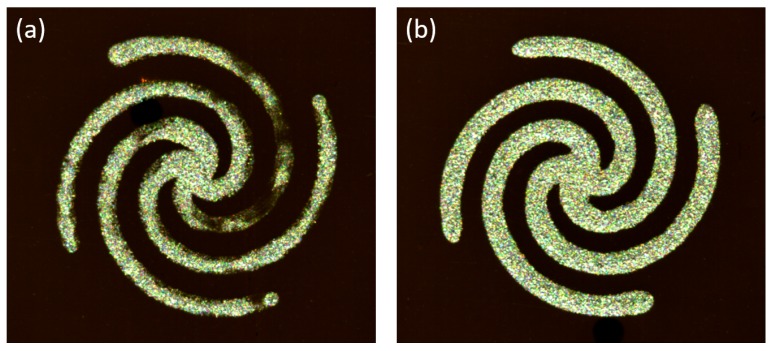
(**a**) Printed spiral image with major print defects including tapered arms, large portions of missing ink, and varied arm width. (**b**) Printed spiral image with near ideal geometry.

**Figure A3:**
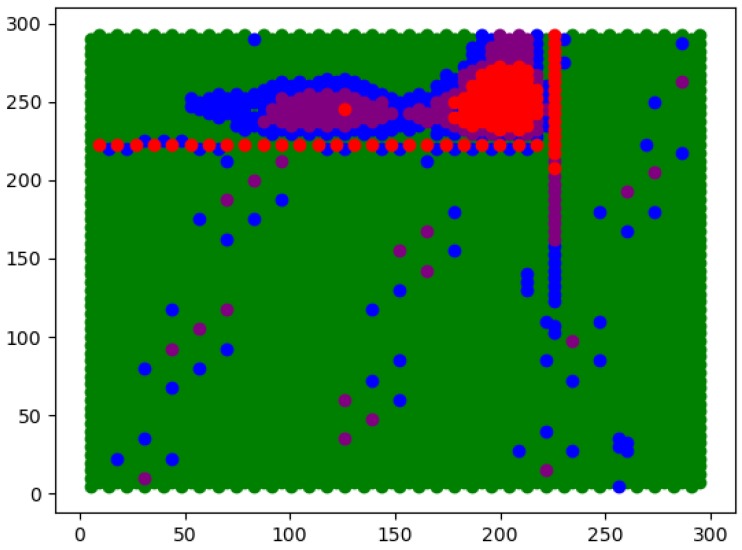
Height map of gantry print stage. Colors indicate standard deviation from the expected height of the point. Expected value (green), 1σ (blue), 2σ (purple), ≥3σ (red).

**Figure A4:**
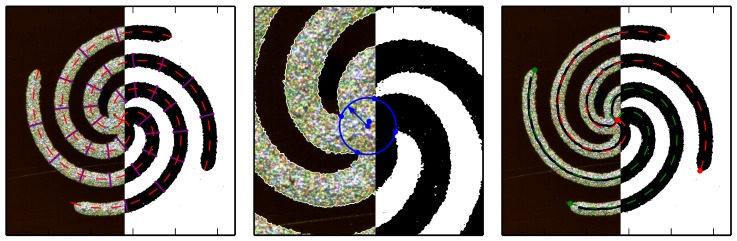
Image processing overlay on a single spiral. (**a**) Width measurement via purple bars perpendicular to the red print path, (**b**) center radius calculation from four blue minimum radius points and circular fit, (**c**) arm length calculation depicted by green starting point arms and red stopping point arms. Note: Images are flipped horizontally in analysis software.

**Figure A5:**
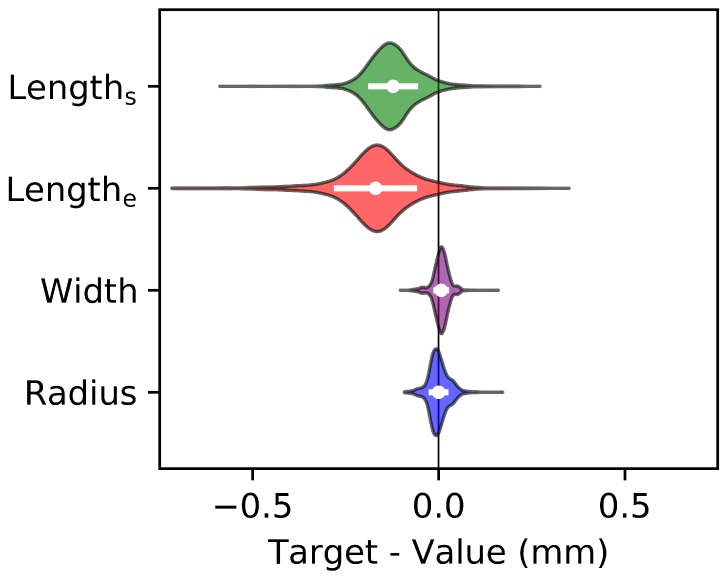
Violin Plots of measured arm length, width, and spiral center radius. The thickness of each violin depicts the probability density of printing a spiral of that value. The white dots indicate the mean while the bars indicate standard deviation. Each parameter is normalized to the target value (x=0).

**Figure A6:**
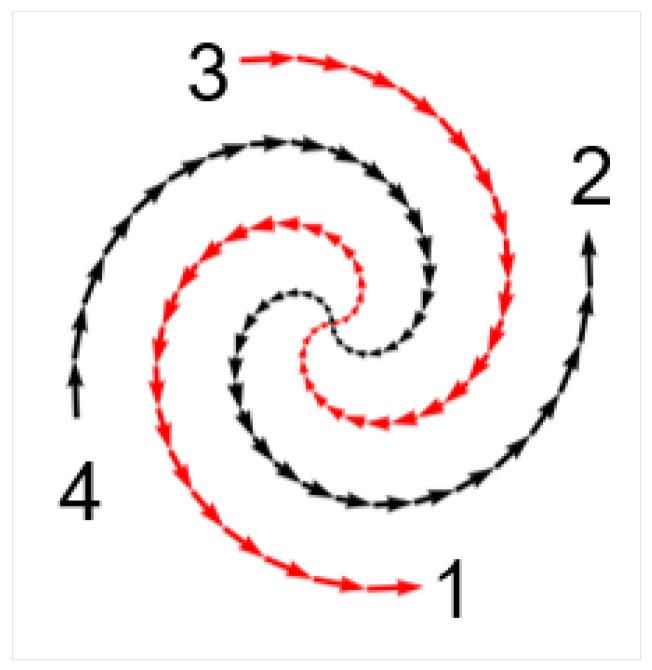
Print path of spiral in extrusion print system. Print path follows 3–1 and 4–2.

**Figure A7:**
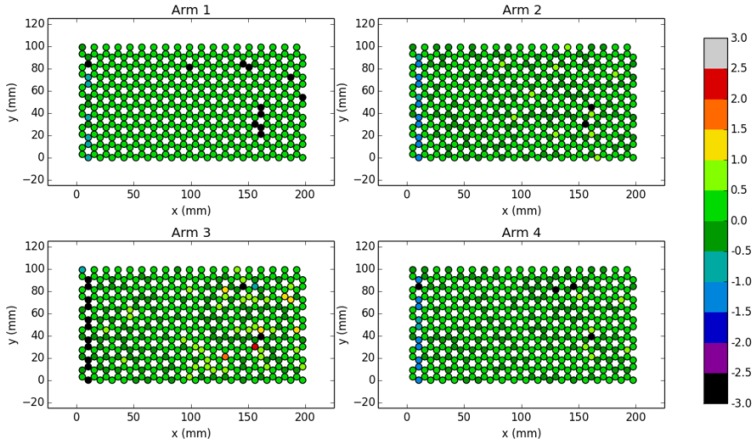
Subset analysis of variances in arm length with respect to target length. Spirals arranged as they are printed.

**Figure A8:**
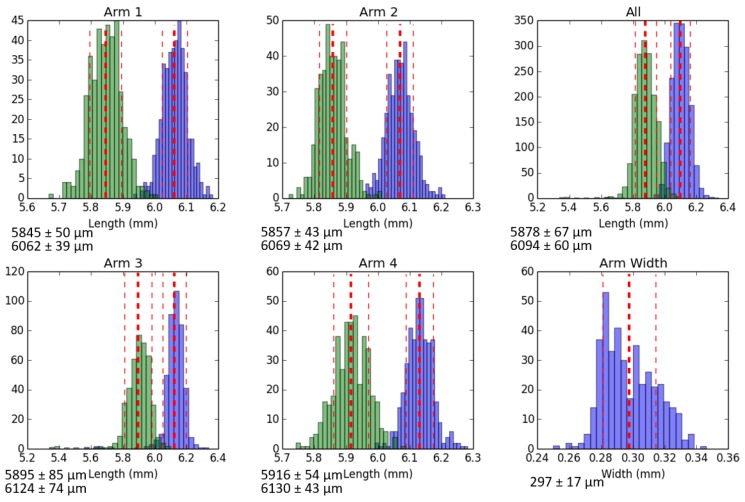
Histogram of dimensions for a subset of 400 spirals. Green bars are arm length measured from the center radius, purple bars are arm length measured from the center of the spiral, red lines are the average and the positive and negative standard deviation.

**Figure A9:**
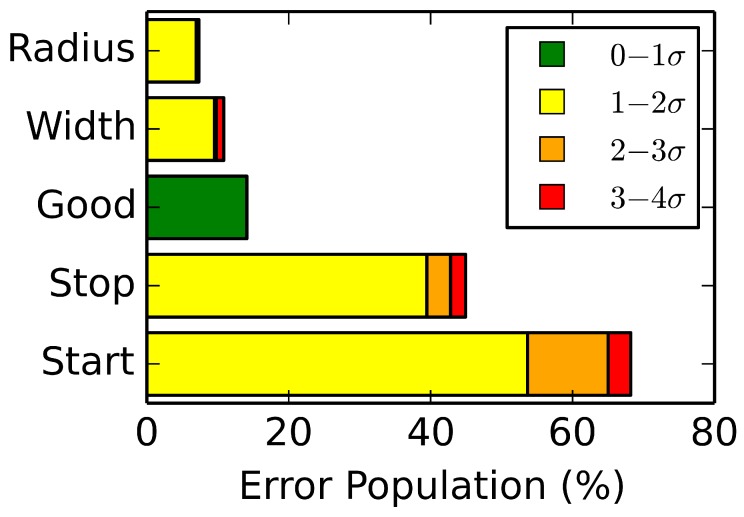
Frequency error count of a single print job. Each color denotes the standard deviation and the bar denotes the percentage of spirals in the set that have a defect (center radius, arm width, stop, and start) and percentage of good spirals is noted in green.

**Figure A10:**
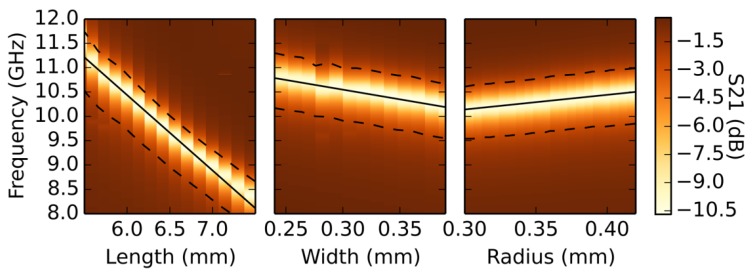
Composite simulated results of spirals with parametric sweep of arm length, arm width, and center radius. The regions outside the dotted black lines imply filtering above −3 dB, the regions within the dotted black lines imply filtering below −3 dB, the solid black lines denote the center frequency of the $S_{21}$ curve.

**Figure A11:**
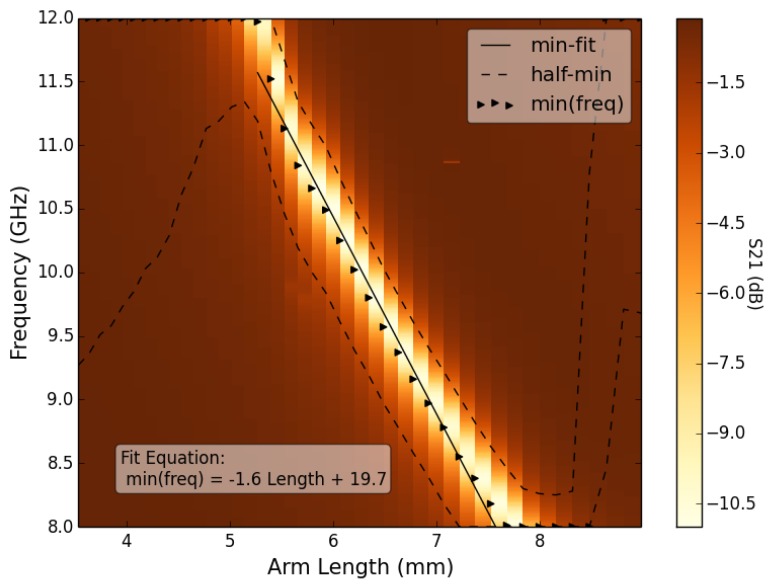
Simulated $S_{21}$ RF Response of Spirals with Varying Arm Lengths. Color indicates $S_{21}$ in dB, triangles indicate the minimum points and dashed lines indicates half of the minimum value.

**Figure A12:**
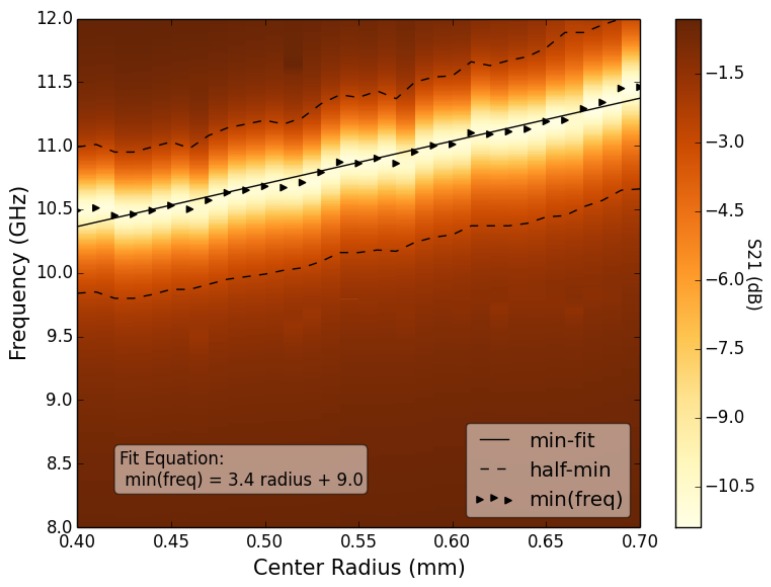
Simulated $S_{21}$ RF Response of Spirals with Varying Center Radii. Color indicates $S_{21}$ in dB, triangles indicate the minimum points and dashed lines indicates half of the minimum value.

**Figure A13:**
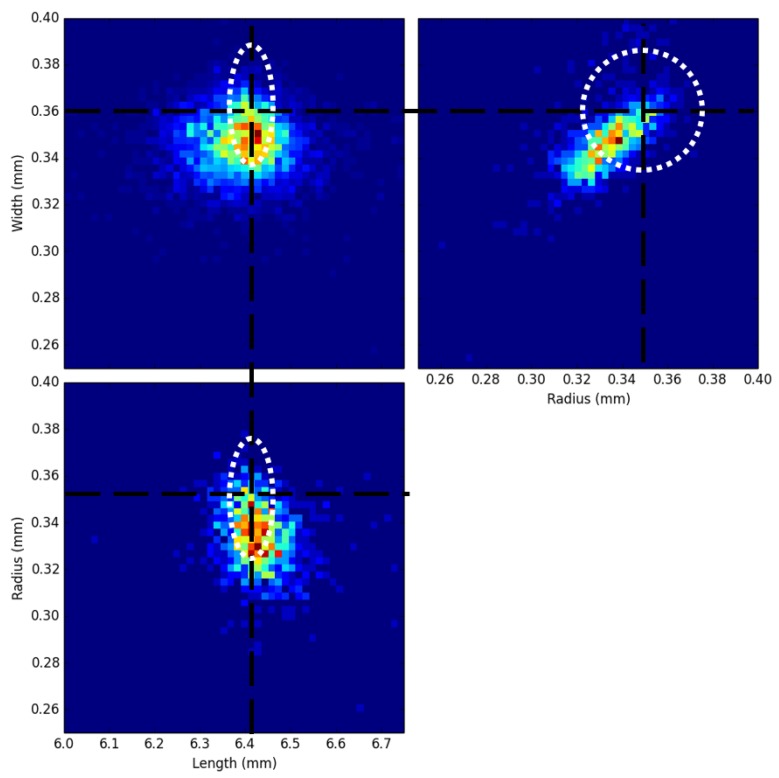
Correlation between arm length, arm width, and center radius. Color scale is normalized to probability density ranging from blue to red. Black lines indicate the target values and the white ellipses indicate the acceptable range.

**Figure A14:**
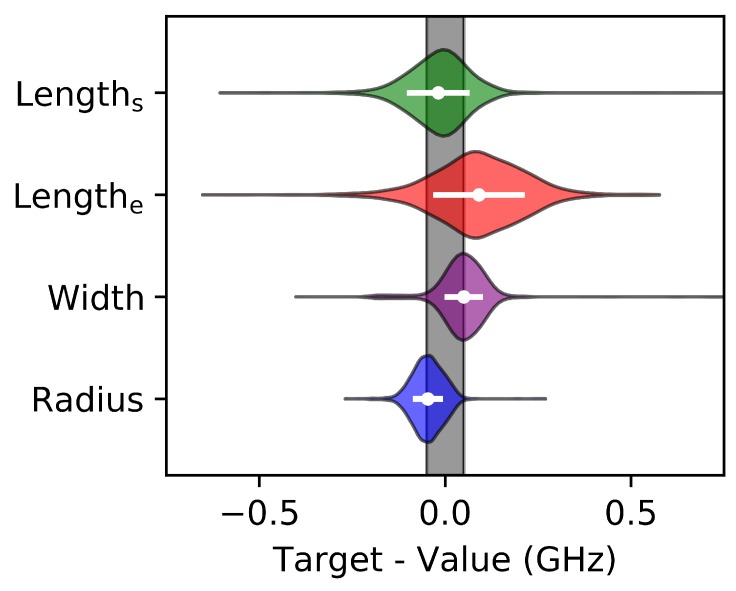
Violin Plots of the measured arm length, width, and spiral center radius. Mapped to frequency space by simulated results. Each parameter is normalized to the target center frequency with an acceptable common band indicated by the shaded grey area.

**Figure A15:**
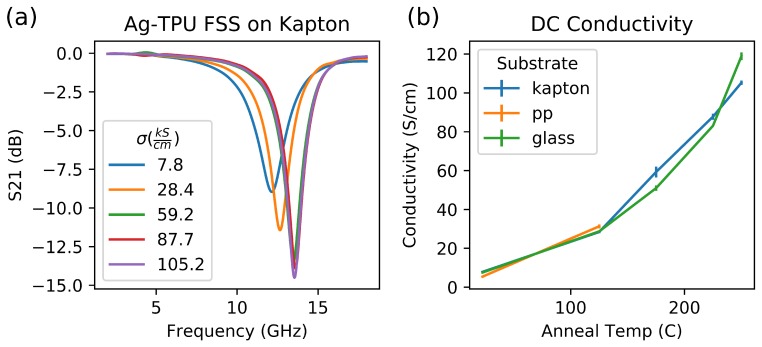
(**a**) Simulated ideal spiral FSS $S_{21}$ response over 0–20 GHz while varying conductivity of Ag-TPU on a Kapton substrate at DC conductivity values, (**b**) Measured DC Conductivity of Ag-TPU on Kapton, polypropylene (PP), and glass as a function of annealing temperature. Note: PP annealing is limited by substrate melting temperature.

## Appendix A. Geometric Analysis of Printed Spirals

Moving and deforming components can have significant impact on frequency response and manufacturing non-uniformities affect performance. To properly take advantage of new additive manufacturing techniques in electromagnetic structures there need to be adequate studies on the impact of process specific defects on RF performance. This appendix serves as a presentation of initial findings in the impact of process specific defects in the case of the spiral FSS presented in this manuscript.

The FSS is a passive meta-material formed of repeated sub-wavelength units and the periodic structure can produce undesired grating lobes. These grating lobes can be minimized by utilizing compact and densely packed elements. However the compact nature can cause additional fabrication concerns which will be discussed below.

The geometric studies in this Appendix are performed on a planar spiral FSS printed on a Kapton substrate using the same spiral design found in Section 2 and print procedures as found in Section 4. A change in substrate results in a shift in center frequency, but the impact of the geometric defects are assumed to be independent of substrate choice.

### Appendix A.1. Print Process Specific Defects

Figure A1 shows a schematic of the syringe print head used in the procedures found in Section 4. Pressure is applied at the top of the syringe and silver ink is extruded through a 200 μm syringe tip. Speed is varied through the print process and has been tuned to minimize defects; however, this extrusion process can introduce defects to the fabricated sample.

Figure A2 shows an example of a spiral with a major defect compared to a spiral with near ideal dimensions. The major defects present in the image can be attributed to multiple parts of the print process, most commonly the missing patches of ink in the arms occur when an air bubble is present in the ink or when the Z-touch probe does not capture the true variance in print stage height with a single reading. A view of the Z-touch probe measurements are seen in Figure A3. Major print defects can have significant impact on the aggregate FSS response when defects are present in large percentages of the population. While major defects can be easily seen, there are many minor defects present in which there are slight variation to the target geometry. These slight variations can come in larger quantities and need to be mitigated to achieve the desired frequency response.

Figure A3 shows the Z-touch probe measurements of substrate height for a single run. Each dot is a single spiral and the colors denote the standard deviation measurement from the anticipated height. In the figure we see pockets of height deviation that are attributed to both substrate warping and print stage design. The lines found at y=225 and x=225 are attributed to the vacuum platens on the print stage that hold the substrate.

### Appendix A.2. Image Processing

During the print process described in Section 4 each spiral is imaged with a high resolution camera post-print. From these images geometric characteristics can be extracted from each spiral. The geometry extraction process is shown in Figure A4. Three key parameters are arm length, measured from the center of the spiral to the edge of the arm, arm width, measured normal to the direction of the arm length vector, and center radius, as defined by the circle placed at the intersection of each arm. These key parameters are studied for their impact on RF response in Appendix A.3.

The analysis process utilizes SciPy [18] and SciKit-Image [19] for geometry extraction and MatPlotLib [20] for data visualization. As the exact *XY*-position of the spiral and its angular orientation are not known at the time of imaging, the algorithm is designed to find the center of the spiral using the SciPy center of mass algorithm which finds the approximate position of the arms by using Scikit-Image’s skeletonize algorithm. The skeleton points are converted from cartesian coordinates into polar coordinates around the center of mass, then a linear fit is applied to one of the arms to determine the orientation of the spiral. From this information the print path is recreated as shown by the red and green dashed lines in Figure A4c and can be traced from the center to the ends of each arm to measure the length. This allows measurement of arm width by tracing along lines normal to the print path until they reach the edge, these normal lines are represented by violet lines in Figure A4a. Multiple arm width measurements are taken to average out the effect of edge roughness and check to see if the arm width is consistent along the length of the arms. While we detected a small increase in width near the center, the width measurements were otherwise constant within the bounds of edge roughness, with respect to length. The center radius is defined by the four edge points, one in each quadrant, closest to the center of mass. The set of edge points is defined by SciKit-Image’s find contours algorithm. A SciPy minimize function finds a center point ($x_{0},y_{0}$) and radius, *r*, which minimizes the fit function described in Equation (A1).

(A1) $$f\left(x_{0},y_{0},r\right)=\sum_{i=1}^{4}\sqrt{{\left(x_{i}-x_{0}\right)}^{2}+{\left(y_{i}-y_{0}\right)}^{2}-r^{2}}$$

The quantity of printing errors can be described in two ways; the accuracy and the repeatability of the parameter. These are generally calculated as the difference between the average observed and expected values, and the standard deviation of the observed values respectively. This is visualized in a set of violin plots in Figure A5. A perfect spiral print would be represented as delta functions at x=0 in the violin plots, implying that all spirals have each parameter equal to the target value. The accuracy is depicted by the *x*-offset of each violin plot, and the repeatability is depicted by the width of the plot. In the context of the FSS functionality, the accuracy is related to the position of the center frequency, while the repeatability influences the Q-factor and bandwidth.

The length and width of each arm is measured independently. This proves important when viewing defects due to starting and stopping the ink extrusion. Figure A6 shows the print path of the spiral. The extruder starts at point 3 and prints to point 1 then moves to point 4 and prints to point 2. The defects result in the length of each arm deviating from the expected parameters. Start and stop defects are minimized by manually adjusting the time between when the pump motion starts and when it stops. In the case of the FSS, we observed that stop defects produced an approximately 50 μm length variation from the arms on which printing started verses when printing stopped (denoted as Length${}_{s}$ for start and Length${}_{e}$ for stop) as seen in Table A1 arms came out shorter than targeted, while the start defects were more successfully controlled. The repeatability of the arm length is poor in many cases, but more poor in the stopping lengths than the starting lengths. The print process is adjusted to account for these errors in later prints from information about previously printed samples.

In contrast, we did not observe a significant variation in arm width between different arms in the print routine and along the length of an arm, thus we present an average value for the width of all arms. The width and radius of the spirals show better accuracy and repeatability as compared to the length of the arms. The camera resolution provides 5 μm per pixel, therefore the accuracy and repeatability of the arm width and center radius are just above the noise threshold.

**Table A1:** Target, Accuracy, and Repeatability Values for a Printed Spiral FSS.

Parameter	Target (mm)	Actual (mm)	Accuracy (μm)	Repeatability (μm)
Length${}_{e}$	5.61	5.44	169	112
Length${}_{s}$	5.61	5.49	122	67
Radius	0.37	0.37	0	26
Width	0.38	0.39	−6	21

Figure A7 shows an example of a study of a subset of spirals in which arm lengths are independently measured and compared among spirals in the lattice. Each arm is measured to preserve information on start and stop defects rather than measuring the average length among the four arms in a single spiral. The colorbar scale refers to the increase or decrease in the millimeter length of the single arm compared to the target length. Each dot is a single spiral in a hexagonal lattice and the variances are denoted by the color of the dot. This visual representation provides feedback on specific areas where print defects occur during a print job. The analysis of geometry spatially in the lattice allows for print process parameters to be tuned prior to printing full samples. This arm length study was conducted as a means of determining where errors occurred in an attempt to mitigate them in later print processes.

Figure A8 shows the distribution of arm lengths and arm widths. The arm length is measured using two methods, one from the center of the spiral (purple) and one from the inner radius (green).

Figure A9 shows an error distribution of a single print job. Each bar shows a defect type and the colors correspond to the standard deviation from the target value for a percentage of spirals.

### Appendix A.3. Impact of Geometry on RF Response

It is difficult to predict the aggregate effect of defects on the frequency response of the FSS a priori. To understand the effect of each defect on RF performance, we performed parametric simulations in Ansys HFSS using a single spiral unit cell with a range of arm lengths, arm widths, and center radii. A representative conductivity of 22,500 Scm was used for the Ag-TPU ink. Over the range we tested, the primary effect of changing a parameter was to shift the frequency response to higher or lower frequencies. While the quality factor of the curves did change over the range of each parameter, the effect was comparatively small. This effect can be seen by the near parallel nature of the −3 dB and −10 dB contour outlines in Figure A10.

The frequency shift due to each parameter is summarized in Figure A10, in which the color scale indicated the $S_{21}$ of each simulated spiral structure, The solid black lines indicate a linear fit to the minimum frequency as a function of each parameter. The fits yielded slopes of −1.55 GHzmm, −3.98 GHzmm, and 3.01 GHzmm for arm length, arm width, and center radius respectively. The fit equations for each parameter are presented in Equations (A2). The slopes indicate that deviations in the width and center radius have a larger effect on the frequency of each spiral than the arm length. This would imply that the width and radius affect the frequency response most strongly; however, as seen in Figure A5 the arm length varies several times more than the radius or arm width indicating that the arm length should widen the frequency response more than the width or radius.

(A2) $$\begin{array}{c} f(l)=-1.55l+19.74\\ f(r)=3.01r+9.24\\ f(w)=-3.98w+11.74 \end{array}$$

A more detailed view of the arm length and center radius impact on RF response can be found in Figure A11 and Figure A12.

It is also worth noting that the geometric parameters are not fully independent when printed using an extrusion method. Figure A13 shows the correlation between arm length, arm width, and center radius of the printed spirals. The color scale in the plot is normalized to probability density ranging from dark blue to red. The target values are indicated by the black dotted lines and the white dotted ellipses indicate the acceptable range of values. This plot shows that the radius and width have a strong correlation with one another while the length does not appear to have a strong correlation with width or radius, this does not imply the length is entirely independent.

As arm width increases, center radius increases as well, and as center radius increases the effective arm length decreases. However, given the opposing trends in Figure A5, it is possible that variations in arm width and center radius will actually cancel each other out, if we assume that the frequency response of each parameter is largely independent of the state of the other parameters. We verified this assumption by running a small set of simulations in which more than one parameter was varied and comparing the simulated frequency response with those predicted by the fits in Figure A10.

The fit equations from the simulated results in Figure A10 map the geometric parameters into the frequency space. In this case, each equation yields a center frequency corresponding to each length, width, and center radius. The result of this mapping is seen in Figure A14 which shows the shift from the target frequency, as calculated from the target parameters. Similar to the position results shown in Figure A5 and Table A1, the arm length has a wider distribution than either the width or radius. Notably, due to the negative slope of the fit equation derived from Figure A10, the relative position of the starting lengths and ending lengths flips across the center axis. This correctly demonstrates that spirals with shorter arms have a higher center frequency.

The translation into frequency space widens the center radius and width distributions more than the arm-length distributions. This is because variations in width and center radius have more than twice the effect on the center frequency per μm. However, the radius and width are expected to still have less of an effect on the center frequency of each spiral due to the high accuracy and repeatability of these parameters. Interestingly, the width and center radius have opposing effects on the center frequency of the spiral. While both the average width and center radius of the spiral populations were slightly smaller than the intended target value the width flips across the target frequency axis. This is of particular interest when considering that the width and center radius are not independent of each other, but rather tend to correlate due to both being governed by the amount of ink extruded. This suggests that errors in width and radius may cancel each other.

A 100 MHz bandwidth is shaded around the target frequency axis in Figure A14 to indicate the region of frequency space in which spirals should have common bandwidth (below −10 dB) with a target spiral. Similar to a common impedance bandwidth, this is the region in which spirals will constructively help filter incoming radiation. Spirals with center frequencies outside of this region may increase the bandwidth, but will not improve the filtering (decrease $S_{21}$) at the target center frequency.

**Table A2:** Center Frequency, Accuracy and Repeatability Values for a Printed Spiral FSS.

Parameter	$f_{\mathrm{center}}$ (GHz)	Accuracy (MHz)	Repeatability (MHz)
Length${}_{e}$	11.31	−263.06	173.92
Length${}_{s}$	11.23	−189.74	104.45
Radius	10.35	0.95	80.38
Width	10.20	27.10	83.73

This analysis should help to understand how each printed feature is affecting the function of the FSS. If most of the parameters shift to one side or the other of Figure A14 then the center frequency will vary from the target frequency. The repeatability of each parameter will impact the bandwidth and quality factor of the FSS. As the distributions widen, the quality factor will decrease. The bandwidth may increase, but this comes at the cost of decreasing filtering power.

### Appendix A.4. Impact of Material Properties on RF Response

In addition to process specific geometric defects, the conductive ink itself has variances in conductivity depending heavily on post-processing conditions. Figure A15 references a small sample study of the impact in conductivity on the center filtering frequency of an ideal spiral geometry. Figure A15b shows the measured DC conductivity of Ag-TPU on three substrates (Kapton, PP, and glass) at five processing temperatures. The PP substrate is limited by its melting temperature (160 ${}^{\circ }$C). Figure A15a shows an ideal spiral simulated in an infinite triangular lattice as described in Section 3. Each curve in Figure A15a shows the simulated frequency response of a spiral on Kapton substrate with varying conductivity following the measured DC conductivity from the post processing conditions in Figure A15b.

The wide range of conductivities from varying post-processing conditions and their corresponding $S_{21}$ responses imply a large impact on anticipated frequency responses in sample printed FSS structures. Varied post-processing temperatures and times can result in a change in conductive paths between silver particles in the ink. Additionally, when annealing elements on a PP substrate, the plastic substrate can deform despite being below the melting point of PP.
